# Diabetes Topics Associated With Engagement on Twitter

**DOI:** 10.5888/pcd12.140402

**Published:** 2015-05-07

**Authors:** Jenine K. Harris, Adelina Mart, Sarah Moreland-Russell, Charlene A. Caburnay

**Affiliations:** Author Affiliations: Adelina Mart, Sarah Moreland-Russell, Charlene Caburnay, Brown School, Washington University in St. Louis, St. Louis, Missouri.

## Abstract

**Introduction:**

Social media are widely used by the general public and by public health and health care professionals. Emerging evidence suggests engagement with public health information on social media may influence health behavior. However, the volume of data accumulating daily on Twitter and other social media is a challenge for researchers with limited resources to further examine how social media influence health. To address this challenge, we used crowdsourcing to facilitate the examination of topics associated with engagement with diabetes information on Twitter.

**Methods:**

We took a random sample of 100 tweets that included the hashtag “#diabetes” from each day during a constructed week in May and June 2014. Crowdsourcing through Amazon’s Mechanical Turk platform was used to classify tweets into 9 topic categories and their senders into 3 Twitter user categories. Descriptive statistics and Tweedie regression were used to identify tweet and Twitter user characteristics associated with 2 measures of engagement, “favoriting” and “retweeting.”

**Results:**

Classification was reliable for tweet topics and Twitter user type. The most common tweet topics were medical and nonmedical resources for diabetes. Tweets that included information about diabetes-related health problems were positively and significantly associated with engagement. Tweets about diabetes prevalence, nonmedical resources for diabetes, and jokes or sarcasm about diabetes were significantly negatively associated with engagement.

**Conclusion:**

Crowdsourcing is a reliable, quick, and economical option for classifying tweets. Public health practitioners aiming to engage constituents around diabetes may want to focus on topics positively associated with engagement.

## Introduction

Diabetes is a major public health problem projected to reach rates as high as 1 in 3 adults in the United States by 2050 (1). Behavior changes, including adopting a healthy diet and increasing physical activity, can decrease the risk of type 2 diabetes and the severity of diabetes-related complications (2,3). There are many online sources for diabetes information, and recent research suggests that a significant proportion of people with diabetes seek health information online (2).

Social media have emerged as popular channels for health information-seeking and sharing; approximately 80% of US adult Internet users have searched online for health information (4,5). Social media are increasingly used by health care providers (5,6) and public health practitioners (7–9) to find and share health information, conduct surveillance, and manage emergency situations.

Social media are unique communication and dissemination tools with interaction, or audience engagement, being a central feature. Social media engagement has been defined as “establishing a connection with others to contribute to a common good” (10). Recent studies suggest public health social media interventions that include opportunities for engagement may have success in prompting small behavior changes (11,12). For example, an intervention linking pedometer use to Facebook encouraged competition among friends for increasing steps taken at work and resulted in a significant increase in steps compared with a control group (13). Engagement with messages sent on Twitter, or “tweets,” is associated with characteristics of both the tweet itself and the sender of the tweet. Specifically, including a hashtag or link in a tweet increases engagement (14). In addition, Twitter user characteristics that include the number of followers, the number of followees (Twitter users being followed), and the age of a Twitter account are also associated with engagement (14). Features of tweets and their senders associated with engagement have been well-studied, but little has been done to identify tweet topics associated with engagement.

Twitter is one of the top 3 social media applications and is used by 19% of all adults and 23% of online adults in the United States (15). Duggan et al (15) found that Twitter was used by more men than women and by more young adults (18 y–49 y) than older adults (50 y–≥65 y). Twitter use rates are higher for non-Hispanic blacks and Hispanics than for non-Hispanic whites. Because diabetes rates are high for men and for Hispanic and non-Hispanic black Americans (1), Twitter may be useful in reaching several groups with high rates of diabetes.

Twitter is an application for “microblogging,” or sending and receiving brief (140 characters or fewer), direct messages (ie, “tweets”) (16). Twitter accounts can be followed by other Twitter users, allowing individuals or organizations to receive and share (“retweet”) messages to their followers, reply to tweets, and mark tweets as a “favorite.” As of October 2013, Twitter estimated that 500 million tweets are sent each day (17). The large volume of tweets presents a challenge for scientists with limited resources in collecting, managing, and analyzing this so-called big data.

Applications such as Amazon’s Mechanical Turk allow the crowdsourcing of small online tasks, also known as Human Intelligence Tasks (HITs). Crowdsourcing is the use of large groups of people, often on the Internet, to do a specific task. HITs are tasks a computer is unable to perform alone; HITs are performed through the use of an open network of workers, also known as “turkers.” A researcher can post HITs that include classification, transcribing, image tagging, and other tasks, which are then completed by turkers, who earn anywhere from half a cent to tens of dollars per HIT completed.

Turkers can work from anywhere in the world; a 2010 study found most turkers reside in the United States (47%) or India (34%). As of April 2014, the percentage of turkers in the United States was 51.5%, and 33% were in India (18). Within the United States, most turkers are male (57%) with a mean age of 32.7 years and are more educated than the general population (73% of the US public has completed at least some college compared with 88% of US turkers) (19). In India, 65% of turkers are male, the average age is 30.5 years, and 81% have a college education (19). Making money is the top motivation for using Mechanical Turk, ahead of other factors such as enjoyment and killing time (20). Evidence regarding the influence of compensation rates is conflicting; early work suggested that low compensation rates (on average $1.60/h) did not affect the quality of completed tasks. However, a recent study found that although compensation did not influence quality for US turkers, turkers from India produced higher quality data for higher compensation (20). Turkers have been used in health-related studies and can be useful in research given their low pricing and speed of service (21).

The widespread use of social media to find health information, including diabetes information, and the potential for social media engagement to influence health behavior presents an opportunity to better understand engagement with diabetes information online. However, the volume of Twitter data accumulating daily presents a challenge for social scientists with limitations on human and financial resources. To address the opportunity and challenge, we sought to 1) examine engagement with diabetes information on Twitter and 2) examine the Amazon Mechanical Turk as a new tool to aid public health researchers working with social media data.

## Methods

### Data collection and classification

As with traditional news sources, Twitter use varies by day of the week (22). To account for this variation, we used a constructed week sampling procedure (23). Specifically, we selected 1 week of randomly selected days (eg, 1 randomly selected Monday, 1 Tuesday) from May and June 2014. We downloaded all tweets that included the hashtag “#diabetes” from each selected day by using the twitteR software package from R (24). The twitteR package allows download of the tweet text and several associated characteristics: screen name of tweet sender, date and time tweet was sent, how many times the tweet was retweeted or favorited (designated a favorite by the reader), and whether the tweet was a “native retweet,” which is a retweet sent by using the Twitter retweet function. We removed native retweets and selected a random sample of 100 tweets from each day. Numerous metrics to capture engagement have been proposed in past research (10,25); we selected 2: favoriting and retweeting. Favoriting is a low-level type of engagement demonstrating agreement with tweet content, whereas retweeting indicates a moderate level of engagement because the retweeter is sharing content with others (12,25).We also collected Twitter user descriptions for each user in the sample who sent a tweet by using the NodeXL Twitter list search function (26).

Three authors (J.K.H., A.M., S.M.R.) reviewed the tweets about diabetes and worked together to develop a classification scheme for each tweet and tweet sender. The classification scheme has 9 topic statements and 3 Twitter user types (Table 1). We entered the classification scheme into the Amazon Mechanical Turk requester system (https://requester.mturk.com/). The topics were entered as a list with checkboxes that allowed turkers to select all topics that applied to each tweet. Twitter user type was entered as a list with radio buttons allowing only 1 type of Twitter user to be selected. The Figure is an example of a HIT from the Saturday data as it would appear to a turker. A HIT included a single tweet for classification.

**Table 1:** Diabetes Topics Associated With Engagement on Twitter: Reliability, Frequency, and Examples of Tweets in Each Tweet Category

Topic and User Characteristic	Example Tweet	ICC (95% CI)	Total Tweets, n (%)	Tweets Favorited, n (%)	Tweets Retweeted, n (%)
**Topic**					
Number or percentage of people with diabetes	@CDCgov estimates that 1 in 3 US adults will have #diabetes by 2050. There’s hope.	.82 (.80–.84)	37 (8.3)	7 (18.9)	7 (18.9)
Diabetes-related joke or sarcasm	My crack dealer #wcw #littledebbie #diabetes @LittleDebbie	.82 (.80–.84)	58 (12.9)	16 (27.6)	3 (5.2)
Diabetes-related event (for example: walk or 5k, conference, awareness month)	This goofy bunch raised over $2,500 to help find a cure for #diabetes. Way to go #TeamReasonRiders! #TourDeCureIndy	.82 (.80–.84)	53 (11.8)	15 (28.3)	23 (43.4)
A person’s success story (for example: good blood glucose, exercise)	Holy Crap!! My blood glucose hasn't been at my goal of 130 in years!! Woo go me:p #diabetes #diabetic	.62 (.57–.67)	37 (8.3)	9 (24.3)	8 (21.6)
A person’s failure or challenge (for example: bad blood glucose, eating candy)	That moment when u eat lunch then realize you forgot to bolus! DOH!! #diabetes #type1 #type2 #organic . . .	.67 (.63–.71)	44 (9.8)	9 (20.5)	3 (6.8)
Children with diabetes	#Diabetes among kids is on the rise #GLV	.83 (.81–.85)	24 (5.4)	6 (25.0)	4 (16.7)
Nonmedical resources for diabetes (eg, recipes, cookbooks, weight loss tips)	Everyone, especially those with #diabetes, need to avoid these 10 processed foods	.70 (.66–.74)	124 (27.7)	26 (21.0)	18 (14.5)
Medical resources for diabetes (eg, new drug, alternative therapy, screening)	Gastric banding: new ammunition in the fight against type 2 diabetes	.72 (.68–.75)	130 (29.0)	23 (17.7)	24 (18.5)
Diabetes-related health problems (eg, heart disease, cancer, amputation, anxiety)	Dr Lane on #diabetes complications: microalbuminuria is a marker for cardiovascular disease risk #APCU2014	.66 (.61–.70)	57 (12.7)	11 (19.3)	10 (17.5)
**Twitter user type**	Example user description	.84 (.81–.86)	NA	NA	NA
Person	Type1 Diabetic, organic enthusiast, stay-at-home dad, blogger	NA	246 (54.9)	54 (22.0)	39 (15.9)
Organization	Therapeutics initiative: providing physicians and pharmacists with up-to-date, evidence-based, practical information on prescription drug therapy	NA	180 (40.2)	37 (20.6)	43 (23.9)
Sender description is blank		NA	22 (4.9)	3 (13.6)	2 (9.1)

Abbreviations: ICC, intraclass correlation coefficient; CI, confidence interval; #, Twitter hashtag; NA, not applicable.

**Figure:**
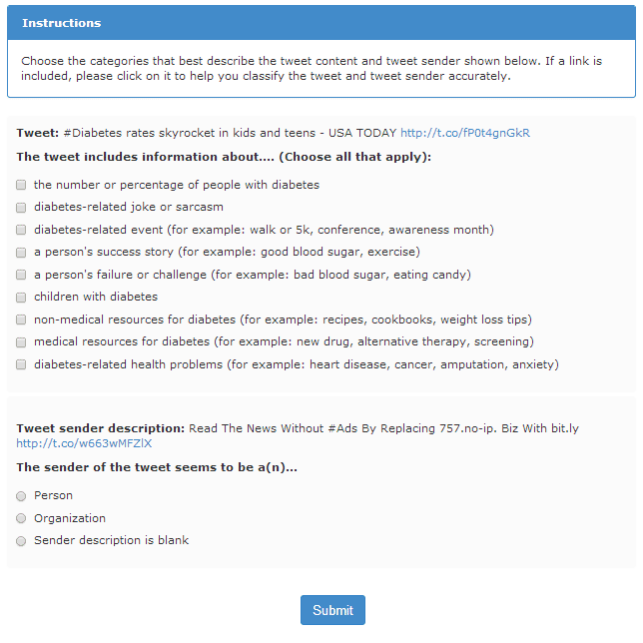
A screen capture of an example tweet and the description of the Twitter user who sent the tweet along with the instructions for classifying the tweet into topic and user categories. At the bottom is the submit button.

**Table:** 

Instructions
Choose the categories that best describe the tweet content and tweet sender shown below. If a link is included please click on it to help you classify the tweet and tweet send accurately.
Tweet: #Diabetes rates skyrocket in kids and teens – USA TODAY
The tweet includes information about . . . (Choose all that apply):
The number or percentage of people with diabetes
Diabetes-related joke or sarcasm
Diabetes-related event (for example: walk or 5k, conference, awareness month)
A person’s success story (for example: good blood sugar, exercise)
A person’s failure or challenge (for example: bad blood sugar, eating candy)
Children with diabetes
Non-medical resources for diabetes (for example: recipes, cookbooks, weight loss tips)
Medical resources for diabetes (for example: new drug, alternative therapy, screening)
Diabetes related health problems (for example: heart disease, cancer, amputation, anxiety)
Tweet sender description: Read The news Without #Ads By Replacing 757.no-ip. Biz With bit.ly
The sender of the tweet seems to be a(n) . . .
Person
Organization
Sender description is blank
Submit

To ensure reliable classification, we followed Hipp et al (27) and requested that each HIT be completed by 4 different turkers. We limited eligibility to turkers who had completed 50 or more HITs with an approval rate of 95% or higher. The classification of 700 tweets 4 times each at $.07 per tweet resulted in a total cost of $196. Amazon charges a fee for use of the Mechanical Turk system. In this case, the settings we selected resulted in a 10% fee, or $19.60, costing a total of $215.60 to classify 700 tweets 4 times each.

### Data management and analysis

To examine reliability of the classification system we used a 1-way random model for absolute agreement (28) to calculate the intraclass correlation coefficient for each topic and user type. Once we determined that the topics and user types were classified reliably, any topic and user type classification selected by 2 or more turkers for a tweet was assigned to the tweet. Finally, although we had a large number of tweets from which to select our daily samples, 66 Twitter users appeared in the data more than once. We examined associations between the number of tweets a user contributed to the data set and the mean number of favorites and retweets per tweet and found no significant association. We also found no significant correlation between the number of tweets a user contributed and the proportion of a user’s tweets in any topic category. In addition, the mean number of tweets in the data set did not differ by user type (ie, organization or individual). To ensure observations were independent, we selected one tweet at random from each of the Twitter users who contributed multiple tweets to the data set. The final sample size was 447 tweets from 447 Twitter users with unique screen names. The final set of tweets was classified by 192 turkers who each coded a median of 5 tweets each (range, 1–86). On average, it took a turker 3 minutes, 26 seconds, to code a single tweet.

We used descriptive statistics and Tweedie regression to examine tweet and Twitter user characteristics associated with engagement. The 2 indicators of engagement, number of favorites and number of retweets, are count variables. Poisson models are often used to model count variables; however, each tweet was favorited a mean of 0.74 times (variance, 52.23), and each tweet was retweeted 0.74 times (variance, 32.03). The magnitude of the variance in relation to the mean violates the Poisson regression assumption that the mean and variance are equal. Having a very large variance in relation to the mean indicates the data are overdispersed. In addition, these data included many zeros for both favoriting (n = 363) and retweeting (n = 367). Tweedie regression accounts for overdispersed count data with a large number of zeros.

We built the regression models in 2 steps. We started with reduced models that included only predictors shown in prior studies to be associated with engagement. Specifically, reduced models included presence of a link in the tweet, the number of followers of the tweet sender, the number of followees of the tweet sender, and the age of the sender’s Twitter account. Although demonstrated as important to engagement, we did not include hashtags as a predictor because all tweets included the hashtag #diabetes as a result of the data collection process. To develop the full model, we then added topic and type of Twitter user to the reduced model.

We used the Aikake Information Criterion (AIC) to determine whether model fit improved from the reduced to the full model. A lower AIC indicates a better-fitting model. In addition, we examined leverage and Cook’s D values to identify and assess outlying and influential values. Analyses were conducted using IBM SPSS version 22 (IBM Corp).

## Results

Tweets were sent by Twitter users with a median of 631.5 followers (range, 7–242,646), and following a median of 613.5 others (followees range, 0–76,742), with accounts open a mean of 1,132 days (standard deviation [SD], 645). The most common diabetes tweet topics were medical resources for diabetes (n = 130, 29.0%) and nonmedical resources for diabetes (n = 124, 27.7%). The least common tweet topic was children with diabetes (n = 24, 5.4%). Tweets about events were most likely to be favorited and retweeted. The percentage of tweets favorited had a small range across tweet topics. The least favorited topic, medical resources for diabetes, was favorited 17.7% of the time, whereas the most favorited topic, diabetes-related event, was favorited 28.3% of the time. The range was much wider for retweeting, ranging from retweets of just 6.8% of tweets about a person’s failure or challenge and 5.2% of a diabetes-related joke or sarcasm to 43.4% of tweets regarding a diabetes-related event. Just over half the tweets were sent by a person (54.9%), 40.2% were sent by an organization, and 4.9% had a blank user description. Interrater reliability was good (0.60–0.74) for half the measures and excellent (0.75–1.00) for the other half. Table 1 shows frequency and reliability for topics,Twitter user type, and example tweets for each category.

There was 1 extreme outlying case for both outcomes and 1 additional outlier for the number of favorites model. The extreme case was an individual with the most followers (n = 262,646) of any of the Twitter users in the data but whose tweets were not favorited and were only retweeted once. The outlier for the favoriting model had the highest value for the number of favorites outcome. Because the 2 cases appeared legitimate, we retained them in the data set.

Reduced and full models were significantly better than null models at explaining the outcomes (*P* < .001). The full models had lower AIC statistics indicating they fit better than the reduced models (Table 2). Significant coefficients indicated that 2 tweet characteristics were positively and significantly associated with being favorited. First, consistent with past research, there was a positive association between a tweet being favorited and the tweet sender having more followers. Second, tweets including information about diabetes-related health problems were positively and significantly associated with being favorited. However, topics negatively and significantly associated with a tweet being favorited were number or percentage of people with diabetes and nonmedical resources for diabetes.

**Table 2:** Tweedie Model Results Predicting the Number of Favorites and Number of Retweets for 448 Tweets Including the Hashtag, “#Diabetes,” Randomly Selected From May Through June 2014

Characteristic	Number of Favorites		Number of Retweets	
	Reduced Model b (SE)	Full Model b (SE)	Reduced Model b (SE)	Full Model b (SE)
**Constant**	−.174 (.379)	−518 (0.588)	−.677 (.426)	−.003 (.590)
**Controls**				
**Followers (100s)**	.002 (.001)^a^	.001 (.001)^b^	.002 (.001)^a^	.001 (.001)^a^
**Followees (100s)**	.003 (.002)	.001 (.003)	.001 (.002)	.001 (.002)
**Account age (100s of days)**	−.072 (.022)^a^	−.026 (.023)	−.049 (.023)^a^	−.019 (.025)
**URL included**	.382 (.379)	.466 (.358)	.771 (.380)^a^	.558 (.400)
**Twitter user type**				
Organization	—	1 [Reference]	—	1 [Reference]
Person	—	.393 (.333)	—	.052 (.322)
No user description	—	−1.286 (1.043)	—	-.085 (.866)
**Tweet topic**				
Prevalence	—	−1.344 (.672)^a^	—	−1.259 (.699)^b^
Sarcasm/joke	—	−.037 (.518)	—	−2.964 (.828)^a^
Event	—	−.454 (.556)	—	−.098 (.506)
Success	—	−.084 (.587)	—	−.416 (.636)
Failure	—	−.948 (.589)	—	−1.153 (.768)
Children	—	−.204 (.749)	—	−.864 (.819)
Nonmedical resources	—	−.839 (.433)^b^	—	−1.441 (.465)^a^
Medical resources	—	−.702 (.443)	—	−.576 (.453)
Health problems	—	1.062 (.454)^a^	—	.388 (.483)
**Model significance^c^**	31.76 (*P* < .001)	64.56 (*P* < .001)	25.37 (*P* < .001)	55.38 (*P* < .001)
**Model fit (AIC)**	853.49	842.70	812.16	804.15

Abbreviations: AIC, Aikake Information Criterion; SE, standard error; —, variable not included in the model.

a *P* < .05.

b *P* < .10.

c Significance calculated using χ^2^.

Likewise, there was a positive and significant relationship between having a large number of followers and retweeting. However, there were negative associations between retweeting and the topics of number or percentage of people with diabetes, diabetes-related joke or sarcasm, and nonmedical resources for diabetes. In addition, although the proportion of tweets retweeted and favorited was highest overall for tweets about events, once other tweet characteristics were accounted for, the event topic was not significantly associated with favoriting or retweeting. Finally, contrary to the results of prior studies, the full models indicated that number of followees, account age, and including a URL did not influence engagement (Table 2).

## Discussion

Through an examination of a sample of tweets about diabetes using crowdsourcing for data classification, we learned 2 things that may aid public health researchers and practitioners working with social media: 1) the Mechanical Turk may be a reliable, quick, and economical way for researchers to code large amounts of complex social media data; and 2) tweet topics may be associated with tweet engagement in public health. Consistent with Hipp et al (27), we found that tweet classification was reliable at the good or excellent level with 4 coders. The total cost associated with tweet classification was low, and the time required to code tweets was minimal, suggesting that crowdsourcing through Amazon’s Mechanical Turk system may be a viable alternative for researchers with limited financial resources to classify large amounts of social media data quickly and reliably.

Research that examined tweet characteristics associated with engagement has primarily relied on methods from computer science including data mining and machine learning. These tools are useful in identifying patterns in social media data related to tweet topic, sentiment (such as sarcasm), and parts of speech. However, the tools have 2 limitations: 1) they require specialized skills not always the purview of social scientists and 2) machine learning algorithms have some limitations in the types of classification they can accurately handle, although methods are increasingly sophisticated and able to handle complex tasks. In contrast, the Mechanical Turk system requires minimal technical skill for use by researchers and provides access to a large population of people with the ability to reliably code many complex topics.

An analysis of tweets classified through Mechanical Turk identified several tweet topics associated with 2 forms of tweet engagement, retweeting and favoriting, which may be explained by tweet topic. Specifically, the topic “nonmedical resources for diabetes” had a negative significant relationship with both favoriting and retweeting. An examination of tweets classified as nonmedical resources indicated that some of these tweets may lack credibility or appear to be spam. For example, this tweet was not favorited or retweeted a single time despite the Twitter user sending the tweet having more than 20,000 followers: “*Learn a Little-Known But 100% Scientifically Proven Way To ERASE Your #Diabetes in 3 SHORT weeks #wellness #health http://t.co/CbaarqLuPu.”*

In addition, retweeting and favoriting were significantly lower for tweets about the number or percentage of people with diabetes, whereas favoriting was higher for tweets about health problems associated with diabetes. This may indicate that Twitter users are engaging with health information specific to their personal health situation but not with general information. Finally, retweeting was significantly lower for tweets that included a diabetes-related joke or sarcasm.

Public health professionals working in diabetes and other areas may wish to consider how Twitter topics influence engagement. Tweet strategies often include guidance on features (eg, hashtags, URLs) to include in a tweet, tweet timing, and other nontopical strategies for increasing engagement. However, our results demonstrated that, controlling for tweet and tweet sender characteristics, tweet topic is influential in whether a tweet is favorited or retweeted.

Our study has several limitations, including the use of a hashtag for data collection. Tweets about diabetes may not contain #diabetes, so we may have missed some important tweets or patterns of relationships. An emerging body of work on hashtag use on Twitter (29) indicates some topics are more likely to be included with a hashtag than others, so use of a hashtag for data collection may have influenced the topics in the tweets we collected. The tweets were collected within 1 to 3 days of being sent. Because Wisemetrics reports that the half-life of tweets is 24 minutes (30), and others report the half life as between 5 minutes and 2.8 hours, it is unlikely the tweets would have accrued a large number of additional favorites or retweets over time. However we cannot rule out that additional favorites or retweets may have occurred given more time. Despite its limitations, our process and findings may be useful to public health researchers studying social media and to public health professionals and organizations that use social media as a way to communicate with constituents about diabetes and other topics.
